# M. Berard’s Case of Intra-Parietal Hernia after a Wound of the Abdomen

**Published:** 1842-11-05

**Authors:** 


*Intra-Parietal Hernia after a wound of the Abdomen.* By M. Berard.—The case detailed in M. Berard's clinical lecture, affords a good example of an accident which is apt to occur not only in penetrating wounds of the abdomen, but in operations for hernia. In the endeavour to force the intestine (which had protruded) back into the abdomen, it was pushed up between the layers of abdominal muscles, and here, in the cavity thus artificially formed, became strangulated. The case was the more perplexing, because, when the intestine was in this position, the finger could be easily passed into the abdomen, and the intestine seemed to be entirely reduced.—*Ibid, from Gaz. des Hôp.* Juin 28, 1842.

